# Investigation on Mn_3_O_4_ Coated Ru Nanoparticles for Partial Hydrogenation of Benzene towards Cyclohexene Production Using ZnSO_4_, MnSO_4_ and FeSO_4_ as Reaction Additives

**DOI:** 10.3390/nano10040809

**Published:** 2020-04-23

**Authors:** Xingai Liu, Zhihao Chen, Haijie Sun, Lingxia Chen, Zhikun Peng, Zhongyi Liu

**Affiliations:** 1 School of Chemistry and Chemical Engineering, Zhengzhou Normal University, Zhengzhou 450044, China; 13275971563@163.com (X.L.); clingxia@vip.163.com (L.C.); 2 Zhengzhou Tobacco Research Institute of China National Tobacco Corporation, Zhengzhou 450001, China; 3 College of Chemistry and Molecular Engineering, Zhengzhou University, Zhengzhou 450001, China; liuzhongyi406@163.com

**Keywords:** Ru, Mn_3_O_4_, benzene, cyclohexene, additives

## Abstract

Mn_3_O_4_ coated Ru nanoparticles (Ru@Mn_3_O_4_) were synthesized via a precipitation-reduction-gel method. The prepared catalysts were evaluated for partial hydrogenation of benzene towards cyclohexene generation by applying ZnSO_4_, MnSO_4_ and FeSO_4_ as reaction additives. The fresh and spent catalysts were thoroughly characterized by XRD, X ray fluorescence (XRF), XPS, TEM and N_2_-physicalsorption in order to understand the promotion effect of Mn_3_O_4_ as the modifier as well as ZnSO_4_, MnSO_4_ and FeSO_4_ as reaction additives. It was found that 72.0% of benzene conversion and 79.2% of cyclohexene selectivity was achieved after 25 min of reaction time over Ru@Mn_3_O_4_ with a molar ratio of Mn/Ru being 0.46. This can be rationalized in terms of the formed (Zn(OH)_2_)_3_(ZnSO_4_)(H_2_O)_3_ on the Ru surface from the reaction between Mn_3_O_4_ and the added ZnSO_4_. Furthermore, Fe^2+^ and Fe^3+^ compounds could be generated and adsorbed on the surface of Ru@Mn_3_O_4_ when FeSO_4_ is applied as a reaction additive. The most electrons were transferred from Ru to Fe, resulting in that lowest benzene conversion of 1.5% and the highest cyclohexene selectivity of 92.2% after 25 min of catalytic experiment. On the other hand, by utilizing MnSO_4_ as an additive, no electrons transfer was observed between Ru and Mn, which lead to the complete hydrogenation of benzene towards cyclohexane within 5 min. In comparison, moderate amount of electrons were transferred from Ru to Zn^2+^ in (Zn(OH)_2_)_3_(ZnSO_4_)(H_2_O)_3_ when ZnSO_4_ is used as a reaction additive, and the highest cyclohexene yield of 57.0% was obtained within 25 min of reaction time.

## 1. Introduction

Cyclohexene is of great significant in modern industry, which can be applied as an intermediate for production of cyclohexanone, adipic acid, caprolactam, nylon 6, nylon 66 and so on [1,2]. Due to the safety, energy conservation, environmental friendly and might result in high carbon atom economy, it has been drawn much attention for the production of cyclohexene via selective hydrogenation of benzene [3]. However, the direct hydrogenation of benzene to cyclohexane is thermodynamically more favorable (∆*G* = −98 kJ mol^−1^, 25 °C) than that obtained by selective hydrogenation to cyclohexene (∆*G* = −23 kJ mol^−1^, 25 °C) [4]. Further, the double bond of cyclohexene is not stable under the hydrogenation conditions, which could be easily continuously hydrogenated into cyclohexane [5]. Therefore, it has always been a goal to develop a catalytic system with relatively high activity and selectivity towards cyclohexene generation from the dynamics point of view [6,7,8].

Among all test catalysts, Ru is considered as the most promising candidate by demonstrating the high selectivity towards cyclohexene synthesis [9]. Surface modification is one of the most effective approaches to enhance the corresponding selectivity, from which promoters have been proven valid [10]. For instance, Cu [11], Co [12,13], Fe [14], Mn [15], Zn [16,17,18,19,20], La [21,22] and Ce [23] were reported to significantly improve the catalytic selectivity towards cyclohexene formation over Ru catalysts. Liu et al. [10] prepared Ru-Ce/SBA-15 catalyst via an impregnation method. They found that Ce existed as Ce^3+^, which may transfer electrons from Ru and enhance the hydrophilicity of the catalyst surface, leading to the improvement of selectivity over Ru catalyst. In addition, Ru-M (M = Fe [24], Mn [24], Zn [24], La [25] and Ce [26]) catalysts were synthesized by Sun et al., from which Fe_3_O_4_, Mn_3_O_4_, ZnO, La_2_O_3_ and CeO_2_ were observed, which could considerably increase the selectivity towards cyclohexene production over Ru catalyst.

On the other hand, in order to improve the selectivity of Ru catalyst towards cyclohexene generation, additives are generally used in the reaction system directly. Struijk et al. [27] thoroughly investigated ZnSO_4_, MnSO_4_, CrSO_4_, CoSO_4_, FeSO_4_, NiSO_4_ and CdSO_4_ as reaction additives, and deemed that these additives could not only increase the hydrophilicity of Ru surface, but also occupy parts of the active sites, which are not suitable for cyclohexene formation. Furthermore, Liu et al. [28] also showed that CdSO_4_ and ZnSO_4_ could be utilized as coadditives. It is suggested that CdSO_4_ could retard the adsorption of cyclohexene, and ZnSO_4_ was able to stabilize the formed cyclohexene and accelerate desorption of cyclohexene from Ru surface. This leads to the inhibition of further hydrogenation of cyclohexene to cyclohexane, thus increase the selectivity towards cyclohexene production.

It is well established that metal catalysts surrounded by metal oxides could effectively suppress the formation of coke and agglomeration of the active component [29,30]. Therefore, in this work, based on the research of promoters for partial hydrogenation of benzene over Ru catalyst, Ru catalyst surrounded by Mn_3_O_4_ was synthesized via a precipitation-reduction-gel method (denoted as Ru@Mn_3_O_4_). And the effect of ZnSO_4_, MnSO_4_ and FeSO_4_ as reaction additives was investigated on the selective hydrogenation of benzene towards cyclohexene.

## 2. Materials and Methods

### 2.1. Chemicals

RuCl_3_·3H_2_O was commercially obtained from Sino-Platinum Co. Ltd. (Kunming, China). ZnSO_4_·7H_2_O, FeSO_4_·7H_2_O, MnSO_4_·H_2_O, NaOH and benzene were delivered from Kemiou Chemical Reagent Co. Ltd. (Tianjin, China). All chemicals were of analytical grade without further purification and distilled water was applied in all experiments.

### 2.2. Preparation of Catalysts

**Scheme 1:**
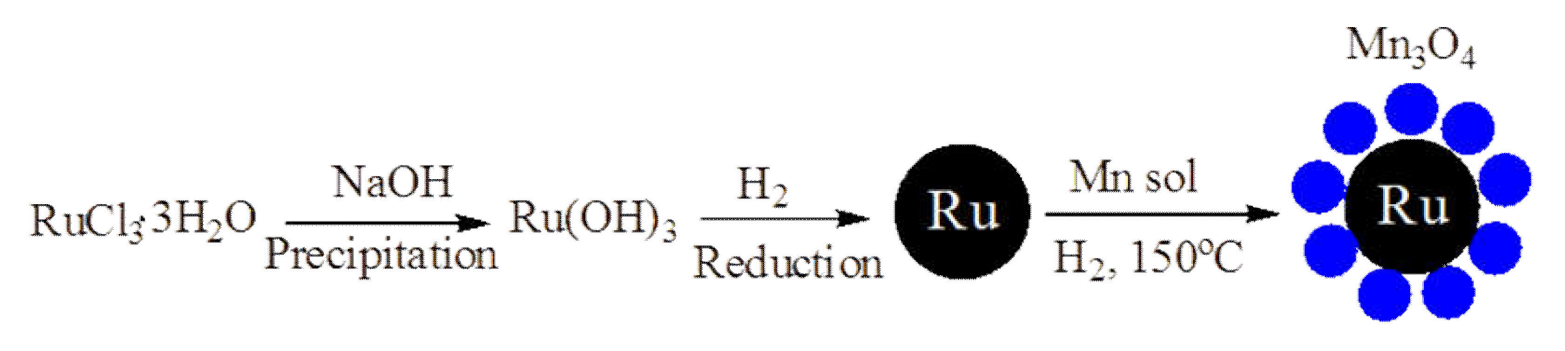
Preparation procedure of Ru@Mn_3_O_4_(*x*) catalysts.

Monometallic Ru catalysts were prepared via the following procedure: 100 mL of 5 wt % NaOH aqueous solution was added in 100 mL of RuCl_3_·3H_2_O solution with concentration of 0.18 mol L^−1^ at 353 K with continuous stirring. After 15 min of stirring, the whole solution was poured in a 1000 mL Hastelloy autoclave (Weihai Chemical Machinery Co., Ltd. Weihai, China) under 5.0 MPa of H_2_ and a stirring speed of 800 min^−1^ at 433 K for 3 h. Then the Ru catalyst was cooled down to room temperature, followed by washing with distilled water until neutral and vacuum-dried. Ru catalyst surrounded by Mn_3_O_4_ was synthesized by the following: certain amount of MnSO_4_·H_2_O and 0.58 g of citric acid was added in 200 mL of distilled water, and the pH was adjusted to 6–7. Subsequently, the aqueous solution heated in water-bath of 333 K to become the Mn_3_O_4_-gel. Then the obtained Ru catalyst as well as Mn_3_O_4_-gel was added in the aforementioned Hastelloy autoclave under 5.0 MPa of H_2_ and a stirring speed of 800 min^−1^ at 433 K for 5 h. After the cooling, washing and vacuum-drying, the obtained material was denoted as Ru@Mn_3_O_4_(*x*), where *x* stands for the molar ratio of Mn to Ru. The preparation procedure of Ru@Mn_3_O_4_(*x*) catalysts is illustrated in Scheme 1.

### 2.3. Catalytic Experimental Procedure

All catalytic experiments were conducted in a 1000 mL GS-1 type Hastelloy autoclave. Of the Ru@Mn_3_O_4_ catalyst 1.8 g and 280 mL of 0.57 mol L^−1^ ZnSO_4_·7H_2_O aqueous solution were added in the autoclave. Then the reactor was purified using N_2_ for 4 times and followed by purification with H_2_ for another 4 times. After the reactor was heated up to 423 K under 5.0 MPa of H_2_ with a stirring speed of 800 min^−1^, 140 cm^3^ benzene and adjusting the stirring speed to 1400 min^−1^ to eliminate the mass transfer limitation. Subsequently, the liquid samples were taken periodically from the reactor every 5 min. All withdrawn samples were analyzed by gas chromatography from Hangzhou Kexiao Chemical Instrument and Equipment Co. Ltd. (Hangzhou, China) equipped with a flame ionization detector (FID). All reagents and products (e.g., benzene, cyclohexene and cyclohexane) were carefully calibrated, and correlation coefficient (*R*^2^) of all compounds is higher than 0.99. And the benzene conversion and selectivity towards cyclohexene were calculated with the obtained peak area with the area normalization method. To avoid the impact of Zn^2+^ on characterization results, catalysts after the reaction were filtered and washed until the filtrate become neutralization and no Zn^2+^ was detected. Then solid samples were dried in Ar flow at 373 K and stored in the ethanol for further characterization.

### 2.4. Procedure Catalysts Characterization

X-ray diffraction (XRD) patterns for the catalysts before and after catalytic reactions were recorded at room temperature using an X’Pert Pro instrument from PAN Nalytical Company (Almelo, The Netherlands). The diffracted intensity of Cu-Kα radiation (λ = 0.15418 nm) was measured at the range of 2*θ* from 5° to 90°, with a step size of 0.03°. Textural properties were given by the Nova 1000e-Physisorption Analyzer from Quantachrome Company (Boynton Beach, FL, USA). All samples were pretreated at 523 K under the vacuum pressure for 2 h before the measurements and the isotherms were taken at 77 K. The specific surface area (S_BET_) was calculated by the Brunauer–Emmett–Teller (BET) model. Furthermore, elemental analysis was gained by X ray fluorescence (XRF) using a S4 Pioneer instrument from Bruker Company (Bruker AXS, Karlsruhe, Germany). In addition, the sample preserved in the ethanol was firstly dispersed with supersonic for 30 min, and then placed on a Cu net-board for TEM analysis using a JEOL JEM 2100 transmission electron microscope (TEM, Akishima, Tokyo, Japan), which combined with energy dispersive spectrometer (EDS) to identify the surface composition of the selected samples. Moreover, the valence state of Ru and Zn on the catalyst surface was explored by X-ray photoelectron spectroscopy (XPS) using a PHI Quantera SXM instrument from UlVAC-PHI Company (Kangawa, Japan). Al Kα (*E_b_* = 1486.6 eV) was selected as the source of radiation and vacuum degree was adjusted to be 6.7 × 10^−8^ Pa. The energy scale was calibrated and corrected for charging using the C1s (*E_b_* = 284.8 eV) line as the binding energy reference.

## 3. Results and Discussions

### 3.1. Effect of Coated Mn_3_O_4_ Amount

**Figure 1:**
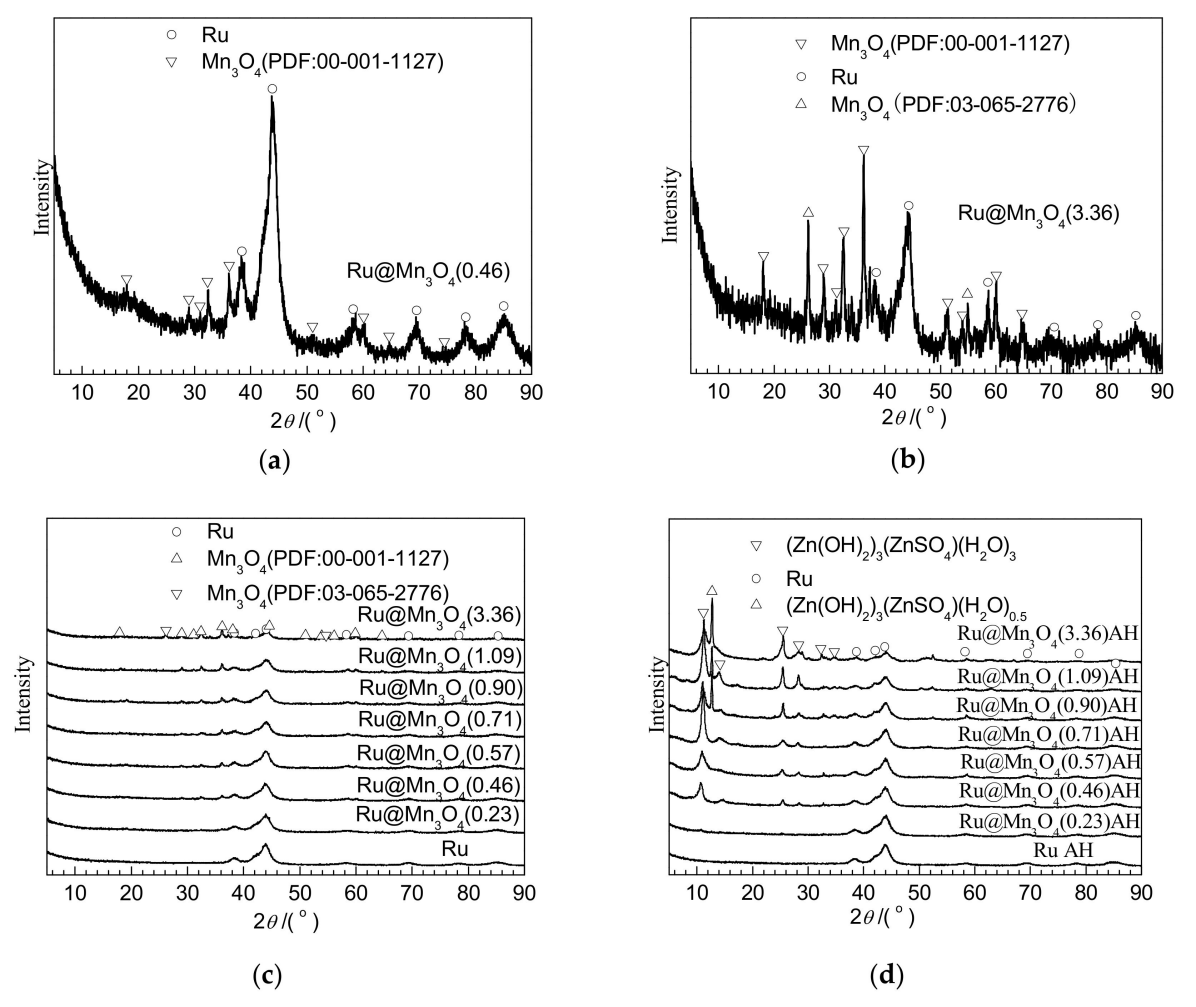
XRD patterns of Ru@Mn_3_O_4_(x) before (**a**–**c**) and after (**d**) catalytic experiments. AH means after hydrogenation reactions.

XRD patterns of fresh Ru@Mn_3_O_4_(*x*) catalyst with different content of Mn_3_O_4_ are illustrated in Figure 1a–c. Characteristic diffractions corresponding to metallic Ru with the hexagonal phase (PDF:01-070-0274) were observed over all measured samples, demonstrating that Ru existed mainly as the metallic state. Furthermore, when the molar ratio of Mn_3_O_4_ to Ru rose up to 0.46, the reflections related to Mn_3_O_4_ of tetragonal phase (PDF:00-001-1127 and PDF: 03-065-2776) started to be shown. This suggests that Ru was successfully coated by Mn_3_O_4_. When the molar ratio of Mn_3_O_4_ to Ru was less than 0.46, no Mn corresponded reflection was observed. This might be mainly due to the fact that the surrounded Mn_3_O_4_ is lower than the detect limitation. Figure 1b shows the Ru@Mn_3_O_4_(*x*) catalyst with different content of Mn_3_O_4_ after catalytic experiments. It can be seen that Ru still existed as the metallic state. On the other hand, as shown in Figure 1b, characteristic diffractions corresponding to metallic Ru with hexagonal phase could also be observed, suggesting that Ru still existed as metallic Ru. Notably, diffractions related to (Zn(OH)_2_)_3_(ZnSO_4_)(H_2_O)_3_ (PDF:00-039-0689) and (Zn(OH)_2_)_3_(ZnSO_4_)(H_2_O)_0.5_ (PDF:00-044-0674) started to appear when the molar ratio of Mn to Ru reached 0.46 and 0.90, respectively. Additionally no Mn_3_O_4_ reflections could be seen. This is mainly attributed to Mn_3_O_4_ being able to react with ZnSO_4_ under the reaction conditions to form (Zn(OH)_2_)_3_(ZnSO_4_)(H_2_O)*_n_* (*n* = 0.5 or 3). It was also confirmed by Sun et al. [15,24] that Mn in Ru-Mn catalysts existed as Mn_3_O_4_, which could react with ZnSO_4_ to generate (Zn(OH)_2_)_3_(ZnSO_4_)(H_2_O)_3_.

Table 1 illustrates the textural properties and crystallite size of pure Ru as well as Ru@Mn_3_O_4_(*x*) before and after hydrogenation reactions. The crystallite size of fresh and spent monometallic Ru catalyst was quite comparable, i.e., 3.6 nm vs. 3.7 nm. This shows that the crystallite of Ru almost had no change during the reaction. Furthermore, the crystallite size of Ru@Mn_3_O_4_(*x*) was larger than that obtained over monometallic Ru no matter before and after hydrogenation reactions, suggesting that the coated Mn_3_O_4_ caused the enhancement of Ru crystallite size. On the other hand, all specific surface area, pore volume and pore diameter over the fresh catalysts tended to decrease with raising the amount of Mn_3_O_4_, implying that the surrounded Mn_3_O_4_ blocked part of the macropores and mesopores of Ru catalysts. More importantly, in comparison to that observed for fresh catalysts, there is a drastically decline on the specific surface area and the pore volume after the catalytic experiments. This suggests that more pores of Ru@Mn_3_O_4_ were blocked by the formed (Zn(OH)_2_)_3_(ZnSO_4_)(H_2_O)*_n_* (*n* = 0.5 or 3) during the reaction.

Composition of pure Ru and Ru@Mn_3_O_4_(*x*) catalysts after catalytic experiments is listed in Table 2. As can be seen, by increasing the amount of coated Mn_3_O_4_, very few Mn species was kept on the Ru surface after the hydrogenation reaction. Unlike what observed for Mn species, an obvious raise of Zn and S content was noticed with enhancing the coated Mn_3_O_4_ content. This implies that Mn_3_O_4_ could react with ZnSO_4_, generating (Zn(OH)_2_)_3_(ZnSO_4_)(H_2_O)*_n_* (*n* = 0.5 or 3), MnSO_4_ and Mn_2_(SO_4_)_3_ (e.g., Equations (1) and (2)). Among all formed compounds, MnSO_4_ and Mn_2_(SO_4_)_3_ were mostly dissolved in the slurry, where (Zn(OH)_2_)_3_(ZnSO_4_)(H_2_O)*_n_* (*n* = 0.5 or 3) was adsorbed on the Ru surface. Therefore, more (Zn(OH)_2_)_3_(ZnSO_4_)(H_2_O)*_n_* (*n* = 0.5 or 3) could be produced by increasing the amount of coated Mn_3_O_4_.

3Mn_3_O_4_+16ZnSO_4_+24H_2_O→4(Zn(OH)_2_)_3_(ZnSO_4_)(H_2_O)_3_↓+3MnSO_4_+3Mn_2_(SO_4_)_3_ (1)

3Mn_3_O_4_+16ZnSO_4_+21.5H_2_O→4(Zn(OH)_2_)_3_(ZnSO_4_)(H_2_O)_0.5_↓+3MnSO_4_+3Mn_2_(SO_4_)_3_ (2)

**Figure 2:**
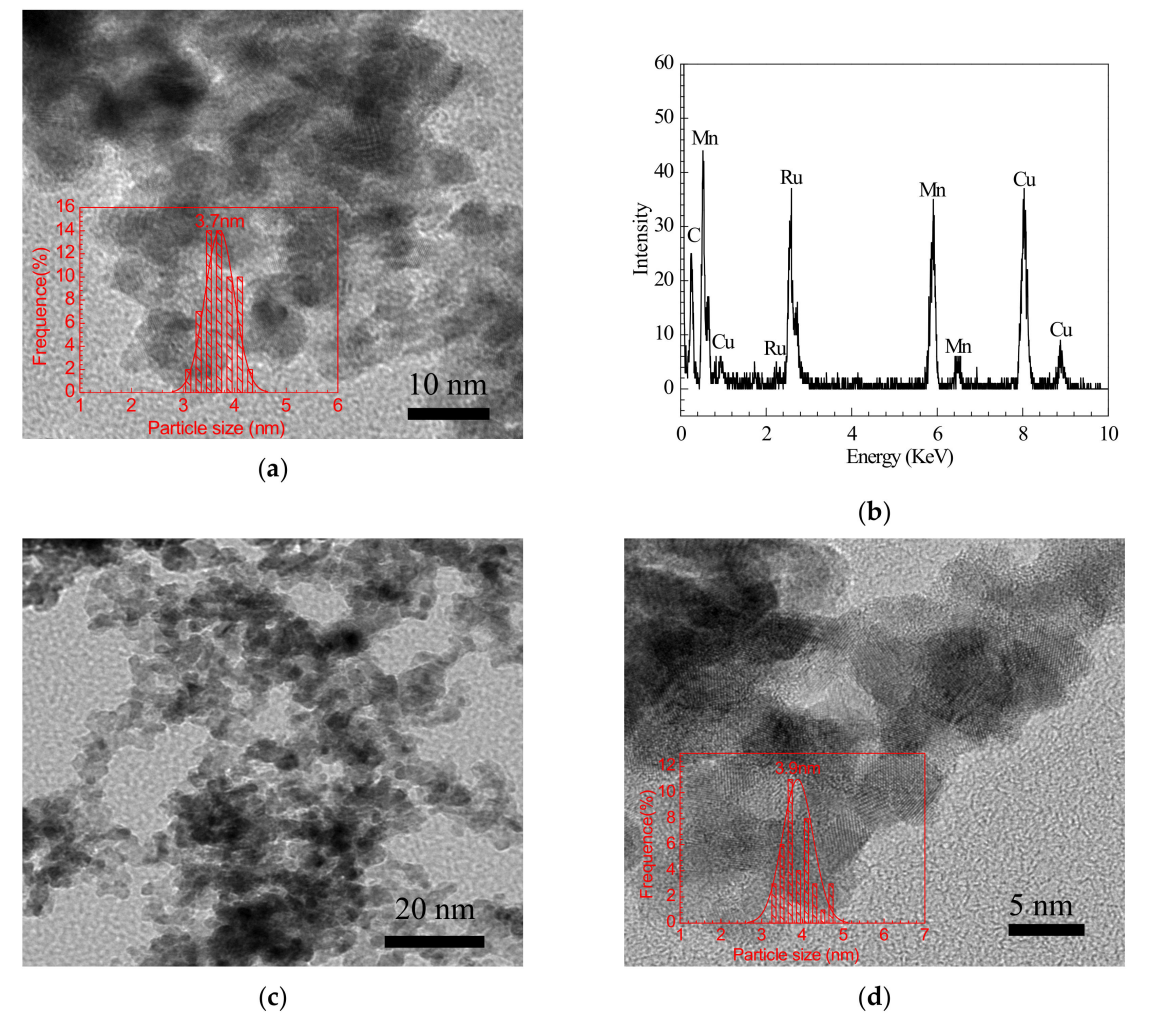

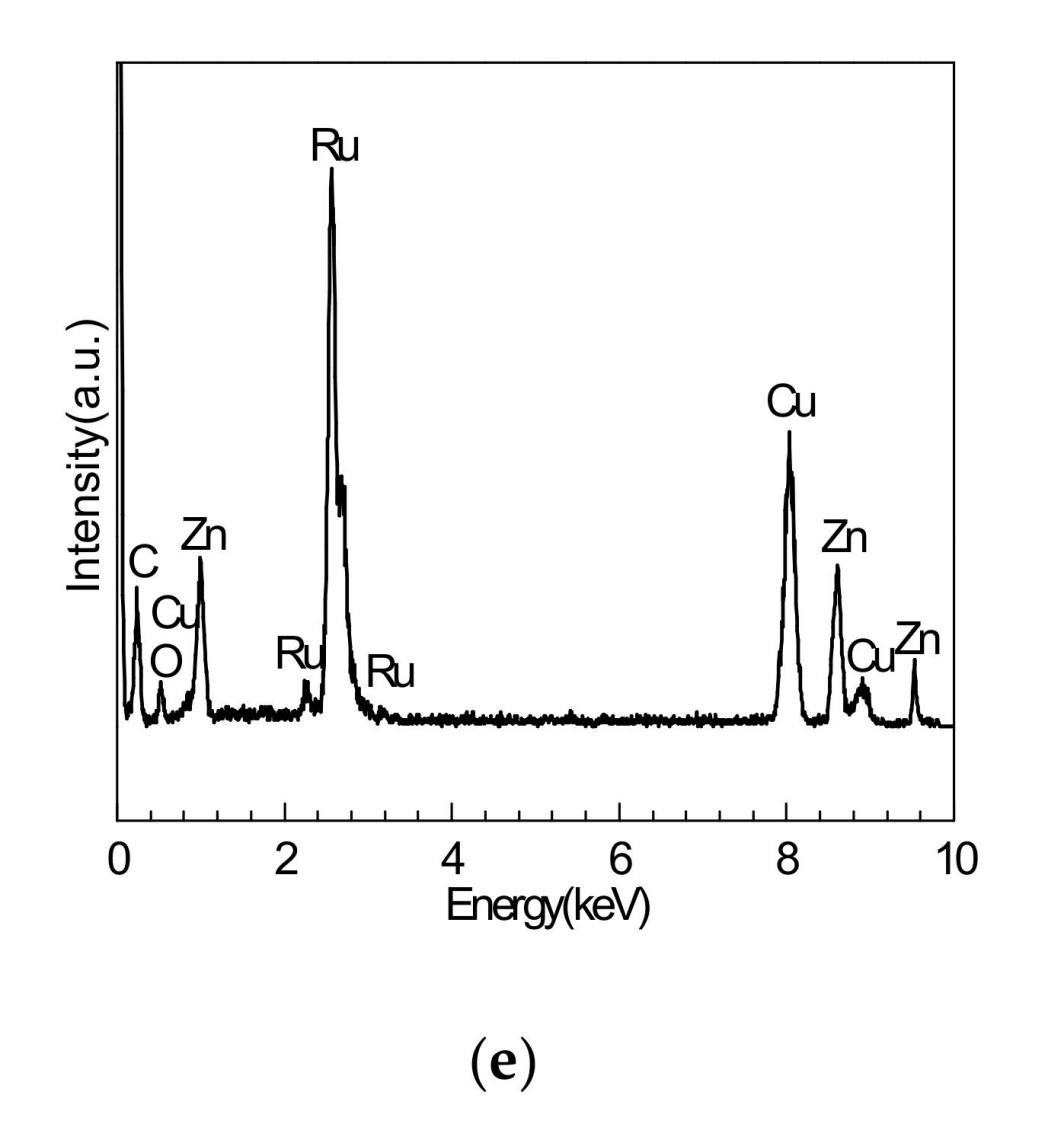
HRTEM (**a**), particle size distribution (insert image) and EDS (**b**) of fresh Ru@Mn_3_O_4_(0.46) as well as TEM (**c**), HRTEM (**d**), particle size distribution (insert image) and EDS (**e**) of spent Ru@Mn_3_O_4_(0.46).

**Figure 3:**
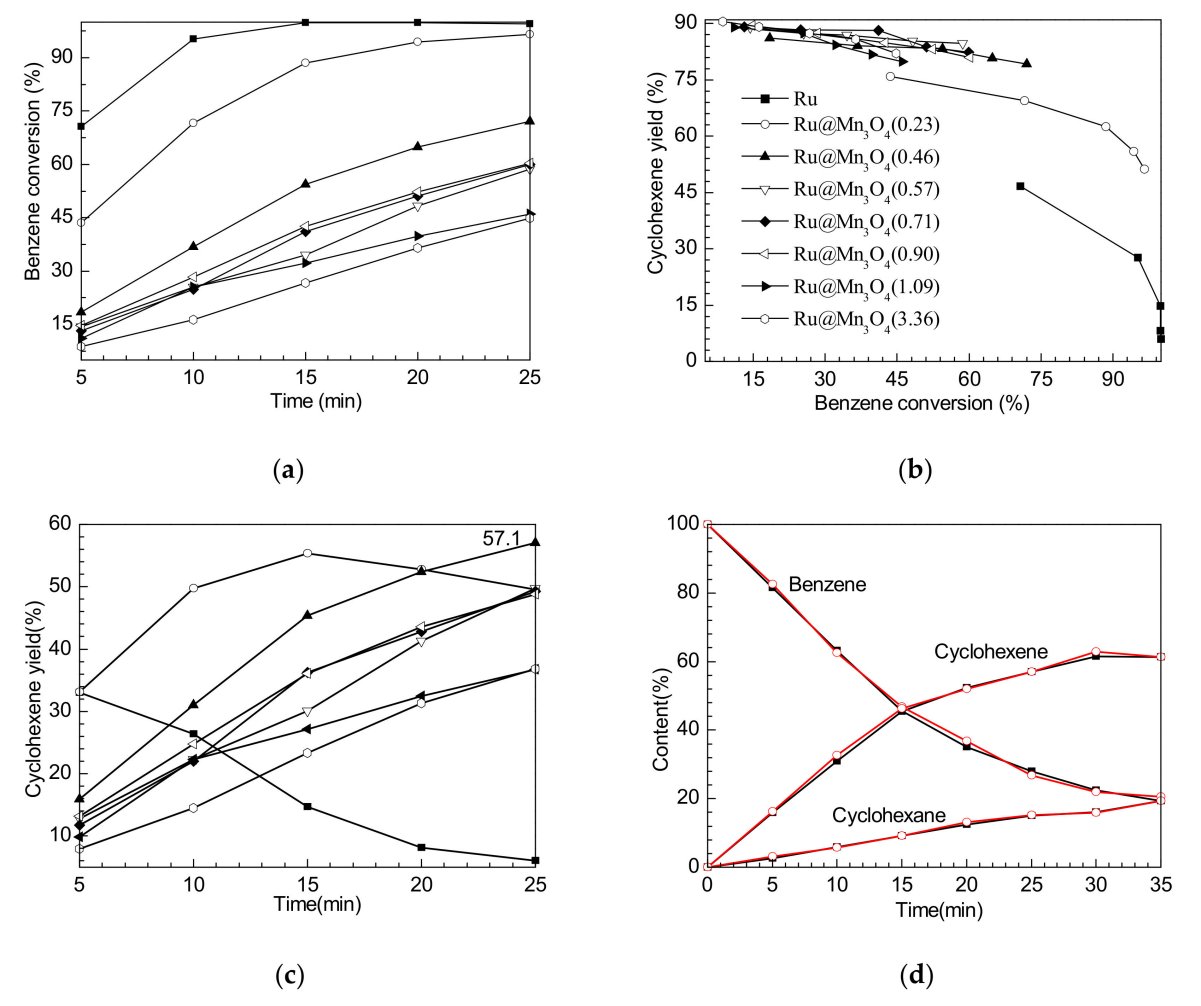
Catalytic activity towards cyclohexene formation from selective hydrogenation of benzene over Ru@Mn_3_O_4_(*x*) catalyst in the presence of ZnSO_4_ with the different molar ratio of Mn to Ru as well as the kinetic curve over Ru@Mn_3_O_4_(0.46) (*m*_cat_ = 1.8 g, *c*_ZnSO4_ = 0.57 mol L^−1^, *v*_H2O_ = 280 mL, *v*_benzene_ = 140 mL, *T* = 423 K, *p*_H2_ = 5.0 MPa). (**a**): Benzene conversion; (**b**): cyclohexene selectivity; (**c**): cyclohexene yield and (**d**): kinetic curve over Ru@Mn_3_O_4_(0.46).

High-resolution TEM (HRTEM) and particle size distribution of fresh Ru@Mn_3_O_4_(0.46) are shown in Figure 2a. It can be observed that fresh Ru@Mn_3_O_4_(0.46) particles were in a circular or elliptical shape, and particle size of the sample was around 3.7 nm. Figure 2b displays the EDS spectrum of fresh Ru@Mn_3_O_4_(0.46). Besides the C and Cu from contaminated CO_2_ and Cu net-board, Ru and Mn were the main elements on the catalyst surface. This again proves that Mn_3_O_4_ is successfully coated on the Ru surface. As can be seen from Figure 2c,d, the spent Ru@Mn_3_O_4_(0.46) still displayed circular or elliptical shape, and its particle size was approximately 3.9 nm. This indicates that the particle shape remained the same, which is in a good agreement with that obtained from XRD results. Furthermore, EDS spectrum of spent Ru@Mn_3_O_4_(0.46) was shown in Figure 3e. In comparison to that observed over fresh Ru@Mn_3_O_4_(0.46) (Figure 3b), reflections of Zn element was demonstrated, while Mn can no longer be seen. This indicates that Mn_3_O_4_ could react with ZnSO_4_, generating (Zn(OH)_2_)_3_(ZnSO_4_)(H_2_O)*_x_* (*x* = 3 or 0.5), which is in a good agreement with XRD results.

Catalytic activity towards cyclohexene formation from selective hydrogenation of benzene over the Ru@Mn_3_O_4_(*x*) catalyst is given in Figure 3a–c. It is obvious that the coated Mn_3_O_4_ significantly improved the selectivity towards cyclohexene formation and diminished the activity towards benzene conversion. When the molar ratio of Mn to Ru was 0.46, 72.0% of benzene conversion, 79.2% of cyclohexene selectivity and 57.0% of yield was achieved within 25 min of reaction time. From the industrial aspect, due to the comparable boiling point, the separation among benzene, cyclohexane and cyclohexene cannot be easily done via a simple distillation procedure. Dimethylacetamide is commonly used as an extractor for the separating purpose [31]. Therefore, selectivity towards cyclohexene production is highly desired in industry to simplify the separation procedure, of which 80% are normally required [9]. Currently, 40% of benzene conversion, 80% of cyclohexene selectivity and 32% of cyclohexene yield meet the basic requirement of cyclohexene production from selective hydrogenation of benzene over a Ru-Zn catalyst in real industry [31]. Thus, Ru@Mn_3_O_4_(0.46) is quite promising as an alternative candidate.

Combined with the characterization results and that reported in the literatures, it is deemed that (Zn(OH)_2_)_3_(ZnSO_4_)(H_2_O)*_n_* (*n* = 0.5 or 3) plays a key role on the formation of cyclohexene. This can be rationalized in terms of the following reasons: (1) A stable complex could be formed between (Zn(OH)_2_)_3_(ZnSO_4_)(H_2_O)*_n_* and cyclohexene, retarding the further hydrogenation of cyclohexene to cyclohexane. Sun et al. [32] reported that ΔG of −618.7 kJ mol^−1^ and −1075.6 kJ mol^−1^ was gained for the complex generated from Zn^2+^ with one and two cyclohexene molecules, respectively. (2) There are several hydroxyl and crystal water in (Zn(OH)_2_)_3_(ZnSO_4_)(H_2_O)*_n_*, generating a stagnant water layer on the catalyst surface. Since the solubility of cyclohexene is lower than that of benzene, it favors desorption of the generated cyclohexene from the catalyst surface through the stagnant water layer, hence suppressing the further hydrogenation of cyclohexene [33]. (3) Part of pores of Ru particles could be blocked by the formed (Zn(OH)_2_)_3_(ZnSO_4_)(H_2_O)*_n_*, resulting in that the hydrogenation of benzene mainly takes place on the surface of catalyst. This further leads to the enhancement of the selectivity towards cyclohexene due to its easy desorption from Ru surface. It was stated by Struijk et al. [33] that cyclohexene generation only occurred on the outer-surface of Ru. (4) (Zn(OH)_2_)_3_(ZnSO_4_)(H_2_O)*_n_* was preferentially adsorbed on the most active sites of Ru [27], leaving the rest active sites with weaker ability for adsorption of benzene and cyclohexene. This would drastically improve the selectivity towards cyclohexene formation, while declining the activity towards benzene conversion. It is in a good agreement with what is observed in this work (Figure 3a–c). (5) There is a strong interaction between Ru and Zn^2+^, which will be further discussed in Section 3.2.

Figure 3d displays the kinetic curve of cyclohexene formation from hydrogenation of benzene over Ru@Mn_3_O_4_(0.46). Of the cyclohexene yield 61.4% was achieved within 30 min of reaction time, which is among the highest yields of cyclohexene ever reported [8,10]. More importantly, it still remained at 61.3% after 35 min of catalytic experiment, describing that (Zn(OH)_2_)_3_(ZnSO_4_)(H_2_O)*_n_* (*n* = 0.5 or 3) could significantly inhibit the further hydrogenation of cyclohexene towards cyclohexane generation. On the other hand, to disprove the homogeneous catalytic effect of Mn salts, a preformed Ru@Mn_3_O_4_(0.46)-ZnSO_4_ catalyst was prepared by adding Ru@Mn_3_O_4_(0.46) in the 0.58 mol/L aqueous solution of ZnSO_4_, followed by a treatment at 433 K under 5 MPa of H_2_ for 30 min. Then the filtered solid was washed and further tested for partial hydrogenation of benzene (red line in Figure 3d). Identical results was achieved over Ru@Mn_3_O_4_(0.46)-ZnSO_4_ in comparison to that obtained over Ru@Mn_3_O_4_(0.46), suggesting no homogeneous catalytic effect of Mn salts in this work. Since (Zn(OH)_2_)_3_(ZnSO_4_)(H_2_O)*_n_* is generated via the reaction between the promoter (e.g., Mn_3_O_4_) and the additive (e.g., ZnSO_4_), it is deemed that the additives are also of great importance for improving the selectivity towards cyclohexene synthesis. Therefore, the effect of additives on selective hydrogenation of benzene over Ru@Mn_3_O_4_(0.46) will be thoroughly investigated.

### 3.2. Effect of Additives

**Figure 4:**
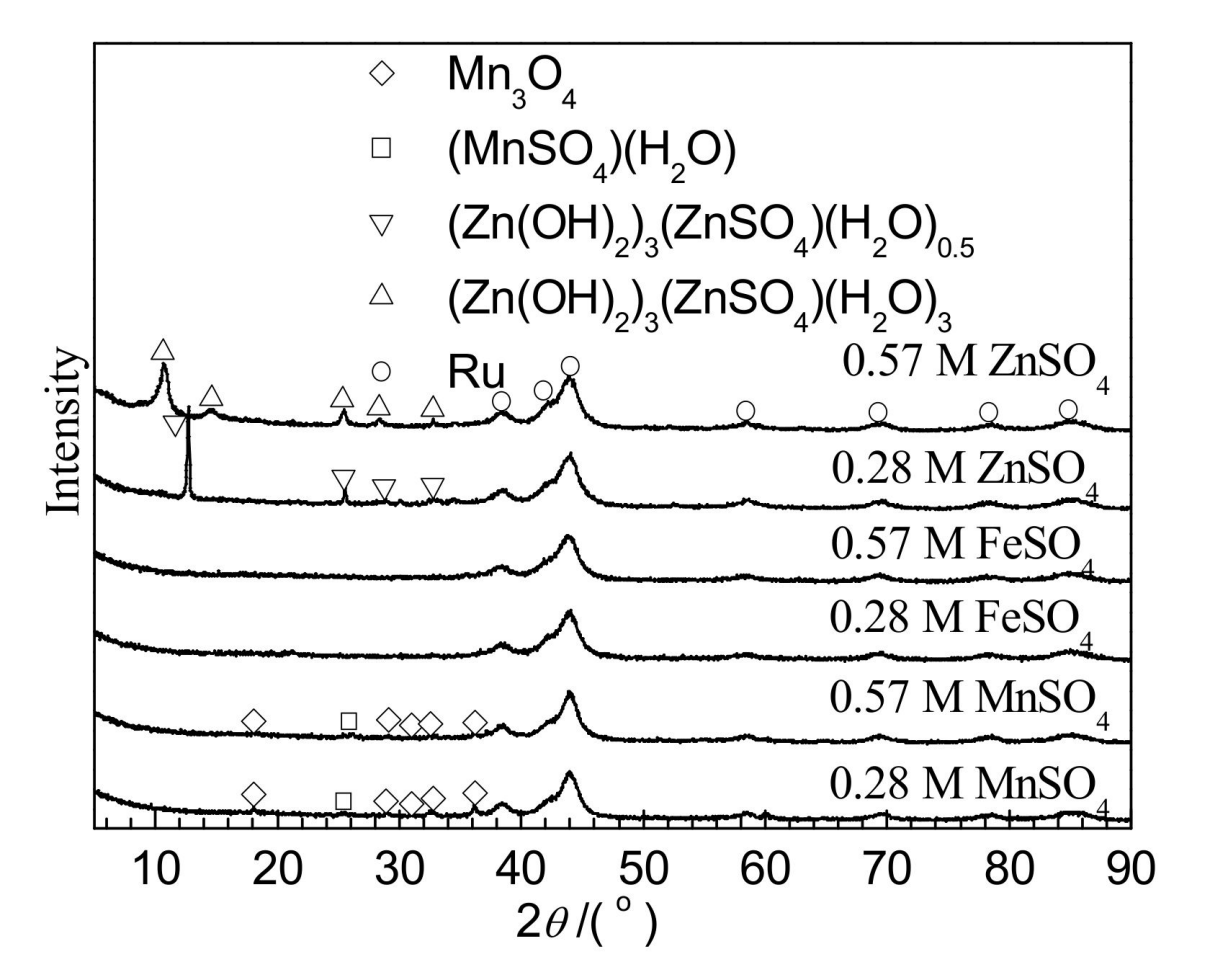
XRD patterns of spent Ru@Mn_3_O_4_(0.46) with 0.28 mol L^−1^ as well as 0.57 mol L^−1^ of ZnSO_4_, MnSO_4_ and FeSO_4_ as additives, respectively.

Figure 4 demonstrates the XRD patterns of Ru@Mn_3_O_4_(0.46) with ZnSO_4_, MnSO_4_ and FeSO_4_ as additives after catalytic experiments. As expected, characteristic diffractions corresponding to metallic Ru were observed over all analyzed samples, indicating that Ru still existed as metallic state with the addition of ZnSO_4_, MnSO_4_ and FeSO_4_ after the hydrogenation reaction. Besides, reflections related to (Zn(OH)_2_)_3_(ZnSO_4_)(H_2_O)*_n_* (*n* = 0.5 or 3) were detected by adding 0.28 mol L^−1^ and 0.57 mol L^−1^ of ZnSO_4_, respectively. This suggests that varying concentration of added ZnSO_4_ causes different content of crystal water. Furthermore, diffractions of Mn_3_O_4_ (PDF:00-001-1127) and MnSO_4_·H_2_O (PDF:00-014-0166) of tetragonal phase were observed when MnSO_4_ was applied as an additive, implying that MnSO_4_·H_2_O can hardly react with Mn_3_O_4_. Additionally, part of the excessive amount of MnSO_4_·H_2_O could be adsorbed on the Ru surface.

**Figure 5:**
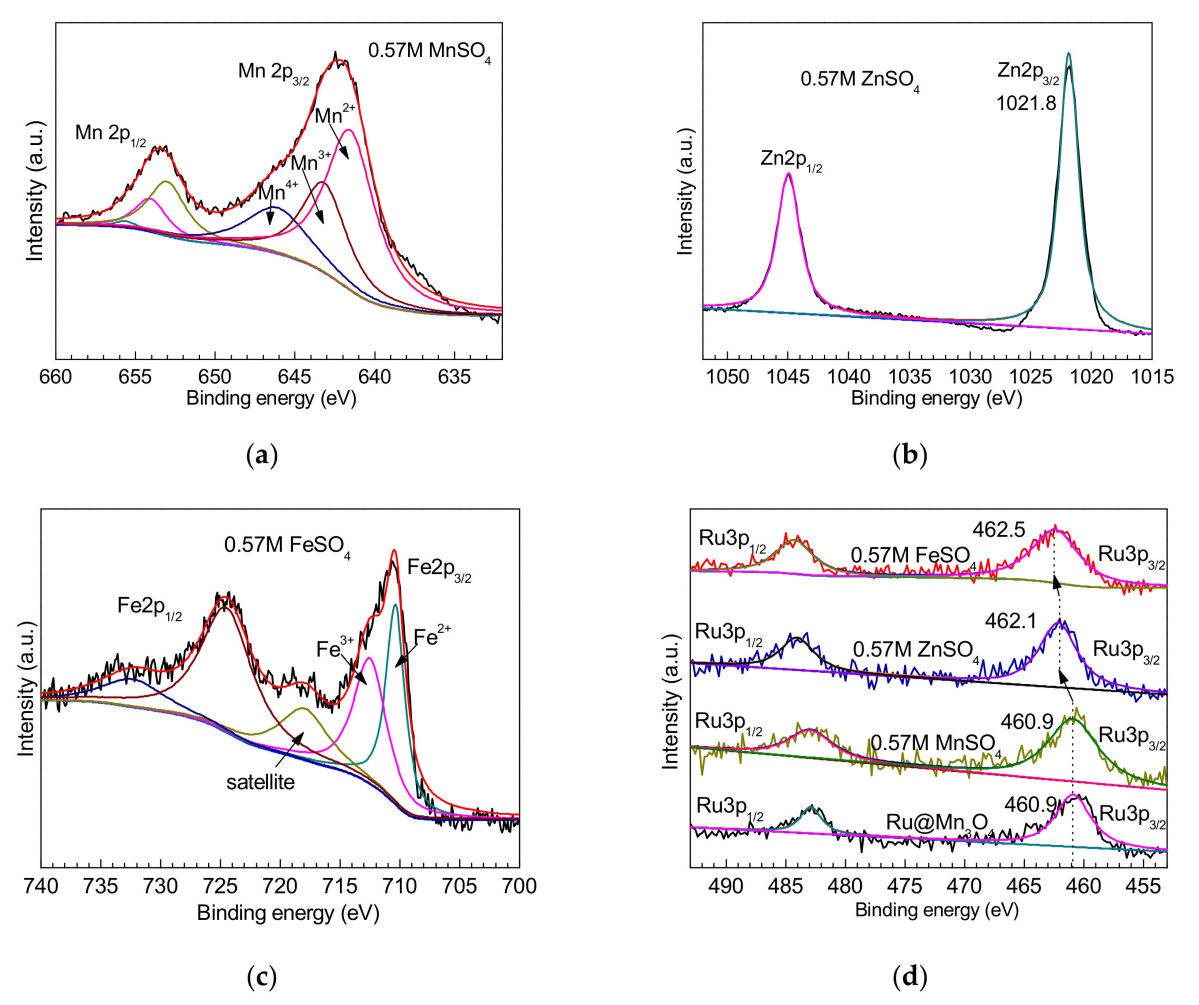
XPS profiles of spent Ru@Mn_3_O_4_(0.46) catalyst with 0.57 mol L^−1^ of ZnSO_4_, MnSO_4_ and FeSO_4_ as additives, respectively. (**a**): Mn 2p of spent Ru@Mn_3_O_4_(0.46) catalyst with 0.57 mol L^−1^ MnSO_4_; (**b**): Zn 2p of spent Ru@Mn_3_O_4_(0.46) catalyst with 0.57 mol L^−1^ ZnSO_4_; (**c**): Fe 2p of spent Ru@Mn_3_O_4_(0.46) catalyst with 0.57 mol L^−1^ FeSO_4_ and (**d**) Ru 3p of the fresh Ru@Mn_3_O_4_(0.46) catalyst and the spent Ru@Mn_3_O_4_(0.46) catalyst with 0.57 mol L^−1^ of ZnSO_4_, MnSO_4_ and FeSO_4_ as additives.

XPS profiles of spent Ru@Mn_3_O_4_(0.46) catalyst with 0.57 mol L^−1^ of ZnSO_4_, MnSO_4_ and FeSO_4_ as additives are demonstrated in Figure 5. As can be observed, three peaks related to binding energy (BE) of Mn2p_3/2_ were observed at 641.7, 643.4 and 646.2 eV, which are attributed to Mn^2+^, Mn^3+^ and Mn^4+^, respectively [34]. Additionally, the peak area obtained over Mn^2+^ and Mn^3+^ was much larger than that given by Mn^4+^, indicating that Mn mainly existed as Mn^2+^ and Mn^3+^ state. Moreover, BE of Zn2p_3/2_ over spent Ru@Mn_3_O_4_(0.46) catalyst was observed to be 1021.8 eV, which is extremely close to that reported for (Zn(OH)_2_)_3_(ZnSO_4_)(H_2_O)_3_ (e.g., 1021. 9 eV) [17]. These are consistent with that obtained from XRD results. Furthermore, BE of Fe2p_3/2_ over the spent Ru@Mn_3_O_4_(0.46) catalyst was shown at 710.4 and 712.6 eV, relating to Fe^2+^ and Fe^3+^, respectively. The small peak at 718.1 eV was attributed to the satellite reflection of Fe^2+^ [35]. This indicates that Fe on the surface of the spent Ru@Mn_3_O_4_(0.46) catalyst existed as Fe^2+^ and Fe^3+^. On the other hand, the BE of Ru2p_3/2_ over spent Ru@Mn_3_O_4_(0.46) without any additives is close to that reported over metallic Ru (461.1 eV) [36]. This implies that electrons were hardly transferred between Ru and Mn_3_O_4_. In addition, the BE of Ru2p_3/2_ over spent Ru@Mn_3_O_4_(0.46) with MnSO_4_ as an additive is the same as that achieved over spent Ru@Mn_3_O_4_(0.46) without any additives, indicating that no electron transfer occurred between Mn and Ru. However, when ZnSO_4_ and FeSO_4_ was applied, the BE of Ru2p_3/2_ over spent Ru@Mn_3_O_4_(0.46) was raised to 462.1 and 462.5 eV, respectively. This suggests that a number of electrons were transferred from Ru to Zn or Fe, generating the Ru^δ+^ species. Additionally, more electrons were transferred from Ru to Fe than that transferred from Ru to Zn.

Composition of spent Ru@Mn_3_O_4_(0.46) with applying 0.28 mol L^−1^ as well as 0.57 mol L^−1^ of ZnSO_4_, MnSO_4_ and FeSO_4_ as additives is listed in Table 3. It can be seen that, by adding MnSO_4_, Ru, Mn and S were detected on the surface of spent Ru@Mn_3_O_4_(0.46), demonstrating the presence of Ru, Mn_3_O_4_ and MnSO_4_. Notably, molar ratio of Mn/Ru and S/Ru was quite comparable, suggesting that a similar content of Mn_3_O_4_ and MnSO_4_ were preserved on the Ru surface remained the same despite the different concentration of applied MnSO_4_. It is worth mentioning that the molar ratio of Mn to Ru drastically dropped when FeSO_4_ was added in comparison to that obtained with addition of MnSO_4_, suggesting that Mn_3_O_4_ could react with FeSO_4_. Meanwhile, a similar trend was observed when different concentration of FeSO_4_ was utilized, where close molar ratio of Mn/Ru, Fe/Ru and S/Ru was obtained. This shows that the amount of generated Fe compounds via the reaction between Mn_3_O_4_ and FeSO_4_ was comparable. Similarly, by addition of ZnSO_4_, the molar ratio of Mn to Ru drastically decreased in comparison to that obtained with addition of MnSO_4_. This is mainly due to the fact that the added ZnSO_4_ could react with Mn_3_O_4_ to generate (Zn(OH)_2_)_3_(ZnSO_4_)(H_2_O)_3_. Interestingly, when 0.57 mol L^−1^ of ZnSO_4_ was applied, the molar ratio of Zn/Ru and S/Ru was 0.2010 and 0.0149, respectively. This is obvious lower than the molar ratio of Zn/Ru and S/Ru by adding 0.28 mol L^−1^ of ZnSO_4_ (0.4997 and 0.0481, respectively). This might be attributed to the hydrolysis of ZnSO_4_, from which the generated acid could react with (Zn(OH)_2_)_3_(ZnSO_4_)(H_2_O)_3_, and thus reduced that content of adsorbed (Zn(OH)_2_)_3_(ZnSO_4_)(H_2_O)_3_ on the Ru surface.

**Figure 6:**
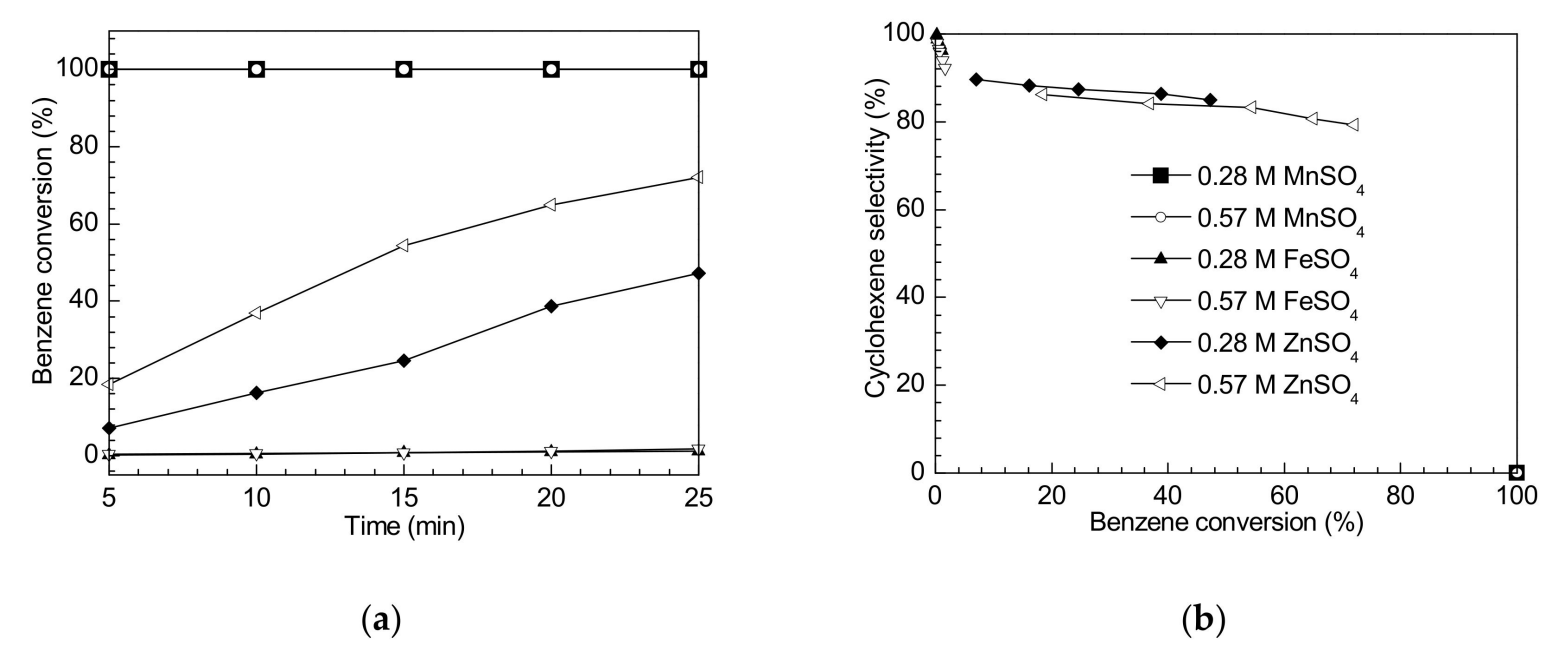
Catalytic activity towards benzene conversion and cyclohexene selectivity over Ru@Mn_3_O_4_(0.46) catalyst with adding 0.28 mol L^−1^ as well as 0.57 mol L^−1^ of ZnSO_4_, MnSO_4_ and FeSO_4_ as additives (*m*_cat_ = 1.8 g, *v*_H2O_ = 280 mL, *v*_benzene_ = 140 mL, *T* = 423 K, *p*_H2_ = 5.0 MPa). (**a**): Benzene conversion and (**b**): cyclohexene selectivity.

Figure 6 shows the catalytic activity towards benzene conversion and cyclohexene selectivity over the Ru@Mn_3_O_4_(0.46) catalyst with adding ZnSO_4_, MnSO_4_ and FeSO_4_ with a concentration of 0.28 mol L^−1^ as well as 0.57 mol L^−1^ as additives. Interestingly, complete conversion of benzene was observed within 5 min when MnSO_4_ (both 0.28 and 0.57 mol L^−1^) was applied, and no cyclohexene was obtained. This can be rationalized in terms that no electron was transferred between Ru and Mn species. Besides, Ru@Mn_3_O_4_(0.46) was also evaluated in the absence of ZnSO_4_, over which complete benzene conversion and 100% of cyclohexane yield was obtained. This further implies that no electron was transferred between Ru and Mn species. On contrary, only 1.2% and 1.5% of benzene conversion as well as 96.2% and 92.2% of cyclohexene selectivity was gained with addition of 0.28 mol L^−1^ as well as 0.57 mol L^−1^ FeSO_4_, respectively. On the other hand, when ZnSO_4_ was applied as an additive, especially with the concentration of 0.57 mol L^−1^, considerably high activity towards benzene conversion (e.g., 72.0%) and selectivity towards cyclohexene (e.g., 79.2%) was achieved. Additionally, 43.7% of benzene conversion and 84.9% of cyclohexene selectivity were obtained over Ru@Mn_3_O_4_(0.46) by using ZnSO_4_ of 0.28 mol L^−1^ as an additive, which was still superior to that gained by using MnSO_4_ and FeSO_4_.

Based on the above discussions, it was confirmed that more electrons were transferred from Ru to Fe than that transferred from Ru to Zn. Therefore, it is concluded that the loss of electrons from Ru plays a key role on the enhancement of selectivity towards cyclohexene formation as well as the decline of activity towards benzene conversion. It is also confirmed that Ru^δ+^ species is crucial in the production of cyclohexene from selective hydrogenation of benzene over Ru-based catalysts [2,6,37]. This can be attributed to the followed two reasons: (1) Less electrons from *d*-orbital of Ru^δ+^ species can be used to active the π-orbital of benzene, leading to that benzene could hardly be directly hydrogenated to cyclohexane [22]. This favors the formation of cyclohexene; (2) the adsorption enthalpy of cyclohexene over Ru^δ+^ is much lower than that over Ru^0^, accelerating the desorption of cyclohexene and thus improves the selectivity towards cyclohexene synthesis. Meanwhile, the adsorption enthalpy of benzene over Ru^δ+^ is also lower than that over Ru^0^, resulting in the decrease of catalytic activity towards benzene conversion [37]. Hence, the highest selectivity towards cyclohexene formation and the lowest activity towards benzene conversion was achieved over Ru@Mn_3_O_4_(0.46) catalyst when FeSO_4_ was added, since the most Ru^δ+^ species were generated. In addition, with increasing the amount of adsorbed (Zn(OH)_2_)_3_(ZnSO_4_)(H_2_O)*_n_* (*n* = 0.5 or 3), more electrons from Ru could be transferred to Zn^2+^. This leads to the increasement of cyclohexene selectivity and decline of benzene conversion. Therefore, due to the lower molar ratio of Zn/Ru by using 0.57 mol L^−1^ ZnSO_4_ than that obtained by applying 0.28 mol L^−1^ of ZnSO_4_, higher catalytic activity towards benzene conversion and lower selectivity to cyclohexene was observed when more ZnSO_4_ was introduced.

## 4. Conclusions

Ru@Mn_3_O_4_ with different Mn_3_O_4_ content was synthesized via a precipitation-reduction-gel method. It was found that 72.0% of benzene conversion and 79.2% of cyclohexene selectivity was achieved after 25 min of reaction time over Ru@Mn_3_O_4_ with molar ratio of Mn/Ru being 0.46. This can be rationalized in terms of the formed (Zn(OH)_2_)_3_(ZnSO_4_)(H_2_O)_3_ on Ru surface from the reaction between Mn_3_O_4_ and the added ZnSO_4_. Furthermore, Fe^2+^ and Fe^3+^ compounds could be generated and adsorbed on the surface of Ru@Mn_3_O_4_ when FeSO_4_ is applied as a reaction additive. The most electrons were transferred from Ru to Fe, resulting in that lowest benzene conversion of 1.5% and the highest cyclohexene selectivity of 92.2% after 25 min. On the other hand, by utilizing MnSO_4_ as an additive, no electrons transfer was observed between Ru and Mn, which led to the complete hydrogenation of benzene towards cyclohexane within 5 min. In comparison, a moderate amount of electrons were transferred from Ru to Zn^2+^ in (Zn(OH)_2_)_3_(ZnSO_4_)(H_2_O)_3_ when ZnSO_4_ was used as a reaction additive, and the highest cyclohexene yield of 57.0% was obtained within 25 min. The effect of reaction additives can be mainly attributed to that the corresponding metal ions adsorbed on the Ru surface and transfer a few electrons from Ru. This leads to the generation of Ru^δ+^ species and thus improves the selectivity towards cyclohexene.

## Figures and Tables

**Table 1:** Texture properties and crystallite size of monometallic Ru as well as Ru@Mn_3_O_4_(*x*) before and after catalytic experiments.

Sample	BET Surface Area (cm^2^/g)^1^	Pore Volume (cm^3^/g)^1^	Pore Diameter (nm)^1^	Ru Crystallite Size (nm)^2^
Ru	59	0.18	10.63	3.6
Ru@Mn_3_O_4_(0.23)	57	0.20	13.74	3.9
Ru@Mn_3_O_4_ (0.46)	41	0.11	10.98	4.1
Ru@Mn_3_O_4_ (0.57)	52	0.15	11.35	4.8
Ru@Mn_3_O_4_ (0.71)	47	0.17	15.79	4.4
Ru@Mn_3_O_4_ (0.90)	54	0.15	11.20	4.6
Ru@Mn_3_O_4_ (1.09)	39	0.10	10.20	4.7
Ru@Mn_3_O_4_ (3.36)	27	0.07	9.48	4.6
Ru AH	56	0.16	10.44	3.7
Ru@Mn_3_O_4_ (0.23) AH^3^	56	0.15	11.03	4.2
Ru@Mn_3_O_4_ (0.46) AH	41	0.11	10.98	4.5
Ru@Mn_3_O_4_ (0.57) AH	33	0.13	15.79	4.6
Ru@Mn_3_O_4_ (0.71) AH	30	0.13	16.92	4.8
Ru@Mn_3_O_4_ (0.90) AH	29	0.11	15.34	4.7
Ru@Mn_3_O_4_ (1.09) AH	26	0.08	12.93	4.2
Ru@Mn_3_O_4_ (3.36) AH	14	0.03	8.72	4.7

^1^ Measured by a N_2_ physisorption instrument. ^2^ Measured by a XRD instrument. ^3^ AH means after hydrogenation reactions.

**Table 2:** Composition of monometallic Ru as well as Ru@Mn_3_O_4_(*x*) after catalytic experiments.

Catalyst	n(Mn)/n(Ru) (mol/mol)^1^	n(Zn)/n(Ru) (mol/mol)^1^	n(S)/n(Ru) (mol/mol)^1^
Ru AH	0	0.0313	0.0026
Ru@ Mn_3_O_4_(0.23) AH^2^	0.0207	0.2010	0.0149
Ru@ Mn_3_O_4_ (0.46) AH	0.0206	0.5085	0.0619
Ru@ Mn_3_O_4_ (0.57) AH	0.0213	0.8369	0.1173
Ru@ Mn_3_O_4_ (0.71) AH	0.0203	1.1733	0.1844
Ru@ Mn_3_O_4_ (0.90) AH	0.0215	1.3726	0.2159
Ru@ Mn_3_O_4_ (1.09) AH	0.0213	2.1539	0.3410
Ru@ Mn_3_O_4_ (3.36) AH	0.0306	4.9727	0.9117

^1^ Measured by an X ray fluorescence (XRF) instrument. ^2^ AH means after hydrogenation reactions.

**Table 3:** Composition of spent Ru@Mn_3_O_4_(0.46) with applying 0.28 mol L^−1^ as well as 0.57 mol L^−1^ of ZnSO_4_, MnSO_4_ and FeSO_4_ as additives.

Additive	*n*(Mn)/*n*(Ru) (mol/mol)^1^	*n*(Zn)/*n*(Ru) (mol/mol)^1^	*n*(Fe)/*n*(Ru) (mol/mol)^1^	*n*(S)/*n*(Ru) (mol/mol)^1^
0.28 M MnSO_4_	0.5006	-	-	0.0503
0.57 M MnSO_4_	0.4728	-	-	0.0507
0.28 M FeSO_4_	0.0195	-	0.4642	0.0374
0.57 M FeSO_4_	0.0172	-	0.4681	0.0321
0.28 M ZnSO_4_	0.0222	0.4997	-	0.0481
0.57 M ZnSO_4_	0.0207	0.2010	-	0.0149

^1^ Measured by an XRF instrument.
